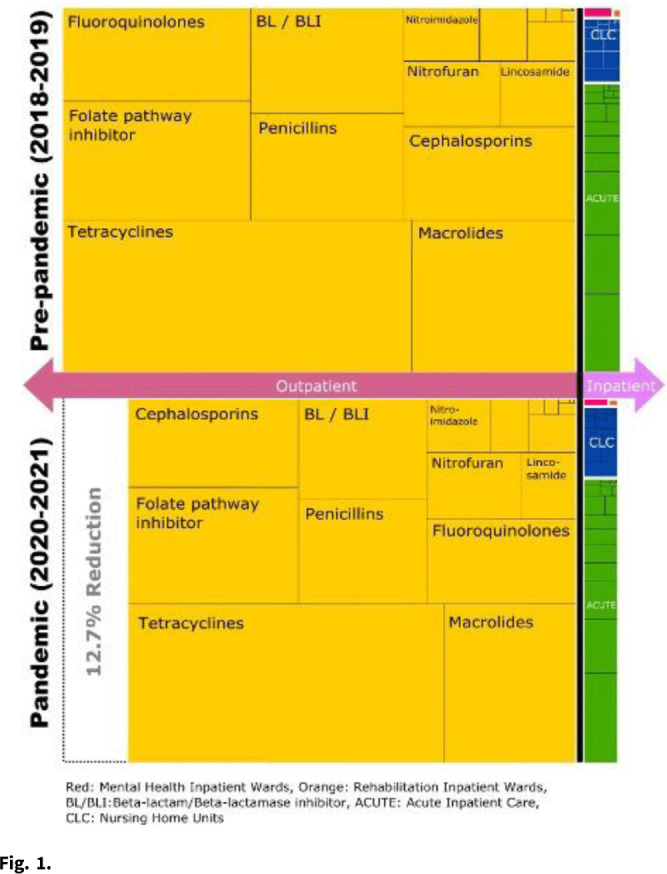# Reduction in outpatient antibiotic utilization: An unintended benefit of the COVID-19 pandemic?

**DOI:** 10.1017/ash.2022.180

**Published:** 2022-05-16

**Authors:** Satoshi Kakiuchi, Eli Perencevich, Daniel Livorsi, Michihiko Goto

## Abstract

**Background:** The COVID-19 pandemic heavily affected healthcare delivery systems in the United States. However, little is known about its impact on overall antimicrobial consumption, especially in outpatient settings. We assessed the impact of the COVID-19 pandemic on antimicrobial consumption in both outpatient and inpatient (acute-care, long-term care, and mental health) settings in the Veterans’ Health Administration (VHA) during the 2 years before and after the start of the pandemic. **Methods:** We conducted a retrospective study for all patients who received care within the VHA from January 2018 to December 2021. We used antibiotic days as the primary outcome measure (days of therapy for inpatient settings and dispensed days supply for outpatient settings), and we obtained data for antimicrobial consumption from the VHA Corporate Data Warehouse. Antibiotics were categorized into classes by the NHSN protocol and included only systemic agents (oral and parenteral). We defined 2018–2019 as the prepandemic period and 2020–2021 as the pandemic period. We compared the relative and absolute difference in antibiotic consumption between the 2 periods. **Results:** Across all periods, 8.3 million patients received care in the VHA, and an average of 28,709,680 antibiotic days were prescribed per year. Overall, 92.9% of all antibiotic days were outpatient and 7.1% were inpatient. Total antibiotic days during the pandemic period decreased by 12.4% compared to the prepandemic period (pandemic period: 53,613,840 and prepandemic period: 61,224,878). This reduction was primarily driven by reductions in outpatient settings (relative reduction: 12.7% and absolute reduction: 7,254,880 antibiotic days over 2 years), but antibiotic days in inpatient settings decreased more modestly (relative reduction: 8.4% and absolute reduction: 356,158 antibiotic days over 2 years) (Fig. 1). When frequently prescribed antimicrobials were categorized by classes, fluoroquinolones and lincosamides showed the largest decreases (fluoroquinolones: 29.2% reduction and lincosamides: 27.2% reduction). Tetracyclines and sulfamethoxazole–trimethoprim had the smallest reductions (5.2% and 11.2%, respectively). **Conclusions:** Compared to the prepandemic period, the pandemic was associated with a substantial reduction in overall antibiotic consumption, especially in outpatient settings, which accounted for 95% of the overall reduction despite being outside the domain of most traditional antibiotic stewardship programs. The impact of the pandemic was most modest in the use of tetracyclines and trimethoprim–sulfamethoxazole and was most prominent in the use of fluoroquinolones and lincosamides. Further studies are required to improve the causal inference between the COVID-19 pandemic and this reduction in antibiotic consumption, as well as its impact on patient outcomes.

**Funding:** None

**Disclosures:** None

**Figure:**